# Construction of a Protective Vaccine Against Lipopolysaccharide-Heterologous *Pseudomonas aeruginosa* Strains Based on Expression Profiling of Outer Membrane Proteins During Infection

**DOI:** 10.3389/fimmu.2018.01737

**Published:** 2018-07-26

**Authors:** Chang Liu, Xiaolei Pan, Bin Xia, Fei Chen, Yongxin Jin, Fang Bai, Gregory Priebe, Zhihui Cheng, Shouguang Jin, Weihui Wu

**Affiliations:** ^1^State Key Laboratory of Medicinal Chemical Biology, Key Laboratory of Molecular Microbiology and Technology of the Ministry of Education, Department of Microbiology, College of Life Sciences, Nankai University, Tianjin, China; ^2^Division of Critical Care Medicine, Department of Anesthesiology, Perioperative and Pain Medicine, Boston Children’s Hospital, Boston, MA, United States; ^3^Department of Anaesthesia, Harvard Medical School, Boston, MA, United States; ^4^Department of Molecular Genetics and Microbiology, College of Medicine, University of Florida, Gainesville, FL, United States

**Keywords:** *Pseudomonas aeruginosa*, vaccine, OprH, outer membrane proteins, immunization

## Abstract

*Pseudomonas aeruginosa* is a ubiquitous opportunistic pathogen, which causes infectious disease in patients with cystic fibrosis and compromised immunity. *P. aeruginosa* is difficult to eradicate because of its intrinsic resistance to most traditional antibiotics as well as acquired resistance mechanisms after decades of antibiotic usage. A full understanding of the *P. aeruginosa* pathogenesis mechanisms is necessary for the development of novel prevention and treatment strategies. To identify novel vaccine candidates, here we comprehensively examined the expression levels of all the known outer membrane proteins in two *P. aeruginosa* strains in a murine acute pneumonia model. OprH was one of the most highly expressed proteins during infection. In addition, OprH is known to be highly immunogenic and accessible by host proteins. Thus, it was chosen as a vaccine candidate. To further identify vaccine candidates, 34 genes highly expressed during infection were evaluated for their contributions in virulence by testing individual transposon insertion mutants. Among them, *fpvA, hasR*, and *foxA* were found essential for bacterial virulence and therefore included in vaccine construction. Immunization with a mixture of FpvA, HasR, and FoxA rendered no protection, however, while immunization by OprH refolded in liposomes elicited specific opsonic antibodies and conferred protection against two lipopolysaccharide-heterologous *P. aeruginosa* strains (PA14 and PA103). Overall, by studying the expression profile of the *P. aeruginosa* outer membrane proteins during infection, we identified OprH as a potential vaccine candidate for the prevention of lung infection by *P. aeruginosa*.

## Introduction

*Pseudomonas aeruginosa* is a Gram-negative opportunistic pathogen which can cause various human infections, especially in immunocompromised and cystic fibrosis patients (1, 2). *P. aeruginosa* is intrinsically highly resistant to a variety of antibiotics, and biofilm formation can further increase resistance by 1,000-fold (3). It is often difficult to eradicate *P. aeruginosa* despite intense antibiotic treatment (4).

Vaccination is an effective strategy to fight against infectious diseases. In the past several decades, enormous efforts have been put into the development of effective vaccines against *P. aeruginosa* (5). In several acute and chronic infection models, animals were protected by immunization with various surface exposed immunogens, including lipopolysaccharide (LPS) O antigen (6), the type III secretion system (T3SS) component PcrV (7), outer membrane protein F (OprF) (8), flagellin (9), and pillin (10). Immunization with these immunogens elicits protective antibodies which mediate opsonophagocytic killing and/or virulence inhibition. Although LPS O antigen-based vaccines confer high levels of protection, the clinical application is limited due to O antigen diversity among *P. aeruginosa* isolates (11, 12). Accordingly, recent studies have been focused on antigens with conserved epitopes.

Multivalent vaccines based on the above antigens showed promising protective efficacy. Immunization with a fusion protein containing OprF-OprI or OprF epitope 8 (OprF311-341), OprI, and type A and B flagellins induced high level of protective IgG and conferred effective protection (13–15). Recently, a bispecific antibody against PcrV and the extrapolysaccharide Psl conferred protection in several murine infection models (16).

Although several vaccines against *P. aeruginosa* infection have entered clinical trials (15, 17–19), no vaccine is currently available for use in humans. Identification of novel candidate immunogens might contribute to the development of effective single- or multi-valent vaccines. Many successful vaccines are based on immunogens with at least one of the following characteristics: high immunogenicity, surface exposure, high abundance, and involvement in virulence (20, 21). *P. aeruginosa* encodes 158 outer membrane proteins (22), of which the expression patterns and functions during infection remain largely unknown. Studies on these genes might provide valuable clues to the understanding of the pathogenesis of *P. aeruginosa* and allow identification of novel vaccine candidates.

Here, we assessed the expression level of each individual outer membrane protein of *P. aeruginosa* in a murine acute pneumonia model. The porin protein OprH was found to be highly expressed during infection and chosen for vaccine construction. Iron uptake proteins, including FpvA, FoxA, and HasR, also showed significantly increase during infection, and can affect the colonization ability of *P. aeruginosa* PA14 in lungs of mice. Vaccination with FpvA, FoxA, and HasR separately or their mixture did not show protective efficacy in murine acute lung infection model. While purified His-OprH also did not have protective efficacy. Vaccination with OprH refolded in liposomes conferred protection against lung infection by two serotype-distinct *P. aeruginosa* strains PA14 and PA103. Together, our study indicates that OprH is a potential candidate for vaccine development against *P. aeruginosa* infection.

## Materials and Methods

### Ethics Statement

All animal studies complied with National and Nankai University guidelines regarding the use of animals in research. All animal experiment protocols have been approved by the institutional animal care and use committee of the College of Life Sciences of Nankai University (permit number NK-04-2012).

### Bacterial Strains and Plasmids

The bacterial strains and plasmids used in this study are listed in Table 1, along with their description and sources.

**Table 1:** Strains and plasmids used in this study.

Strain or plasmid	Description	Reference or origin
***Pseudomonas aeruginosa***		
PAO1	Wild type	(23)
PA14	Wild type	(24)
PA103	Wild type	(23)
PA14Δ*oprH*	PA14 with oprH deletion	This study
PA14/pUCP20-GFP	PA14 with GFP expression	This study
PA14Δ*oprH*/pUCP20-GFP	PA14ΔoprH with GFP expression	This study

***E. coli***		
E1447	pE926 transferred into BL21(DE3)	This study
E1414	pE1409 transferred into BL21(DE3)	This study
E1386	pE1328 transferred into BL21(DE3)	This study
E1787	pE1787 transferred into BL21(DE3)	This study

**Plasmids**		
pE926	fpvA gene of PA14 were cloned into pET28b	This study
pE1409	hasR gene of PA14 were cloned into pET28b	This study
pE1382	foxA gene of PA14 were cloned into pET28b	This study
pE1724	oprH gene of PA14 were cloned into pET28b	This study

### Preparation of Bacterial Inocula for *In Vivo* Challenge Experiments

Bacteria were grown overnight at 37°C in LB. The bacteria were diluted 1:100 in fresh medium and grown to an OD_600_ of 1.0. For intranasal challenge experiments, bacteria were washed with phosphate-buffered saline (PBS) and diluted to the indicated concentrations. The concentrations of bacteria were confirmed by plating and enumeration.

### Murine Acute Pneumonia Model

Six-week-old female BALB/c mice were purchased from Vital River (Beijing, China). Mice were anesthetized with an intraperitoneal injection of 7.5% chloral hydrate (100 µL per mouse) and then inoculated intranasally with 20 µL *P. aeruginosa* strain PAO1, PA14, or PA103 at the indicated bacterial concentrations.

For colonization assays, 12 h postinfection, mice were sacrificed CO_2_, lungs were isolated and then homogenized in 1% peptone, and bacterial numbers were determined by serial dilution and plating. For survival assays, mice were monitored for 5 days after infection.

### Quantitative Real-Time PCR Assay

To examine bacterial gene expression levels during infection, mice were sacrificed by CO_2_ at 3 or 6 h postinfection. Bronchoalveolar lavage fluid (BALF) was obtained by cannulation of the trachea followed by two instillations of 1 mL sterile PBS with 0.5 mM EDTA. 50 µL of the BALF was used for bacterial enumeration, while the remaining BALF was centrifuged and the pellets were immediately resuspended in 200 µL TRIzol reagent (Invitrogen). Total RNA was isolated as instructed by the manufacturer and further purified with an RNA cleanup kit (Tiangen Biotech). For *in vitro*-grown bacteria, overnight cultures of bacterial cells were diluted 1:100 into fresh LB medium and grown to an OD_600_ of 1.0. Total RNA was isolated with an RNeasy Minikit (Tiangen Biotech).

cDNA was synthesized by a PrimeScript Reverse Transcriptase (TaKaRa) with random primers. The cDNA was mixed with 5 pmol of forward and reverse primers (Table S1 in Supplementary Material) and iQSYBR green Supermix (Bio-Rad). Quantitative real-time PCR was conducted using a CFX Connect Real-Time system (Bio-Rad). The 30S ribosomal protein gene *rpsL* was used as an internal control (25).

### Expression and Purification of Proteins From *E. coli*

*fpvA, foxA, hasR*, and *oprH* were cloned from genome of *P. aeruginosa* strain PA14 by PCR. His-tagged fusion proteins were constructed in pET28a and the resulting plasmids were introduced into *E. coli* BL21(DE3). Expression of the proteins were induced by IPTG (1 mM). Bacteria were harvested by centrifugation at 4,000 × *g* for 20 min and lysed by sonication on ice in buffer B (100 mM NaH_2_PO_4_, 500 mM NaCl, 8 M urea, 10 mM imidazole, pH 7.2). The lysate was centrifuged at 10,000 × *g* for 30 min at room temperature. The supernatant was mixed with Ni-NTA agarose (Qiagen) and incubated at room temperature for 1 h. The lysate-resin mixture was loaded into an empty column and washed twice with buffer B containing 25 mM imidazole. The protein was eluted by buffer B containing 500 mM imidazole, followed by further purification by molecular sieve (GE). To remove urea and imidazole, proteins were extensively dialyzed in PBS with reducing concentrations of urea (4, 2, and 1 M) and finally in PBS. Protein concentration was measured with the Bradford method (Bio-Rad).

### Preparation of FpvA, FoxA, and HasR

After dialyzed into PBS, the concentration of FpvA, FoxA, and HasR were 0.2, 0.5, and 0.2 mg/mL, respectively. These proteins were mixed with same volume of curdlan (20 mg/mL) and used for mice immunization. To construct the Fe receptor mix, equal amount of His-FpvA, His-HasR, and His-FoxA were mixed together and dialyzed into PBS. The final concentration of supernatant was 0.9 mg/mL. The proteins were mixed with the same volume of curdlan (20 mg/mL) before immunization.

### OprH Refolding

Refolding of OprH in 1, 2-dihexanoyl-*sn*-glycero-3-phosphocholine (DHPC) micelles was performed as described previously (26). Briefly, 0.4 mM OprH in buffer B containing 500 mM imidazole was diluted 10-fold into the refolding buffer (20 mM Tris–HCl, 5 mM EDTA, and 0.6 M l-arginine, pH 8.5) with 3% DHPC (Avanti Polar Lipids Inc.). The mixture was incubated at 37°C for 72 h and then dialyzed against 2.5 L of 20 mM Tris–HCl, 5 mM EDTA, and 50 mM KCl at pH 8.5 for 20 min at room temperature. The solution was concentrated by an ultrafiltration device (Millipore), and the buffer was exchanged with an exchange solution (25 mM Na_3_PO_4_ and 50 mM KCl at pH 6.0) by ultrafiltration. The final concentration of the refolded OprH was approximately 7 mg/mL.

### Immunization of Mice

Mice were immunized three times with refolded OprH plus curdlan (10 mg/mL) in PBS intranasally at weekly intervals. Three weeks after immunization, the sera of mice were obtained or the survival assay was conducted.

### Enzyme Linked Immunosorbent Assay (ELISA)

96-well plates were coated with purified OprH at 0.1 mg/mL in coating buffer (15 mM Na_2_CO_3_ and 35 mM NaHCO_3_, pH 9.6) overnight at 4°C. For the whole cell ELISA, PA14 and the Δ*oprH* mutant were grown overnight at 37°C in LB. The bacteria were diluted 1:100 in fresh medium and grown to an OD600 of 1.0. 1 × 10^8^ bacteria were washed once by PBS and resuspended in 1 mL PBS. 50 µL bacteria suspension were added in each well of 96-well ELISA plate and dried at 56°C. 200 µL cold methanol were added to incubate at room temperature for 15 min. The plates were washed three times with PBST (PBS containing 0.05% Tween 20) and blocked with 1% BSA for 2 h at 37°C. Then 100 µL of serially diluted serum from immunized mouse was added into each well of the plate and incubated for 1 h at 37°C. Each well was washed three times with PBST, followed by addition of 100 µL diluted HRP-conjugated goat anti-mouse IgG antibody and incubation at 37°C for 1 h. Then, each well was washed three times with PBST. 200 µL horseradish peroxidase color development solution (Beyotime) was then added into each well and incubated at room temperature. 50 µL 2 M H_2_SO_4_ was added to end the reaction, and OD_450_ were measured using spectrophotometer.

### Phagocytosis Assay

The phagocytic uptake by bone marrow-derived macrophage (BMDM) was performed as previously described (27–30). BMDMs were differentiated as previously described (31). 2 × 10^5^ BMDMs were seeded into each well of a 24-well plate 24 h before incubation with bacteria. Wild-type PA14 or the Δ*oprH* mutant was grown to an OD_600_ of 0.6–1.0. The bacteria were collected and washed once with HBSS. 5 × 10^7^ bacterial cells suspended in 50 µL HBSS were incubated with 20 µL heat-inactivated (56°C for 20 min) mouse serum for 30 min at 25°C. Then the bacteria were washed twice with HBSS and resuspended in 1 mL HBSS, of which 50 µL were added into each well and incubated for 40 min at 37°C. Gentamicin was added to each well at 250 µg/mL and incubated for 10 min to kill the extracellular bacteria. The cells were then washed three times with pre-warmed HBSS and lysed in 1 mL cold sterile water. The intracellular bacteria were numerated by serial dilution and plating.

To observe the phagocytosis of bacteria, BMDMs were seeded on poly-d-lysine coated cover slips. The cells were incubated with PA14 or Δ*oprH* containing a green fluorescent protein (GFP) overexpression plasmid (pUCP20-GFP). After treatment with gentamicin for 10 min, cells were washed twice with PBS and fixed with 4% paraformaldehyde for 20 min at room temperature. Microscopy slides were covered with cover slips using mounting medium and observed with a fluorescence microscope.

### Statistical Analyses

All analyses were performed using Prism software (GraphPad Software, La Jolla, CA, USA). Survival data were analyzed with the log-rank test and the Gehan–Breslow–Wilcoxon test. Parametric data were analyzed by Student’s *t*-test (for two-group comparisons) or ANOVA with Dunnett’s multiple comparison test.

## Results

### Expression Profile of *P. aeruginosa* Outer Membrane Proteins During Lung Infection

**Figure 1:**
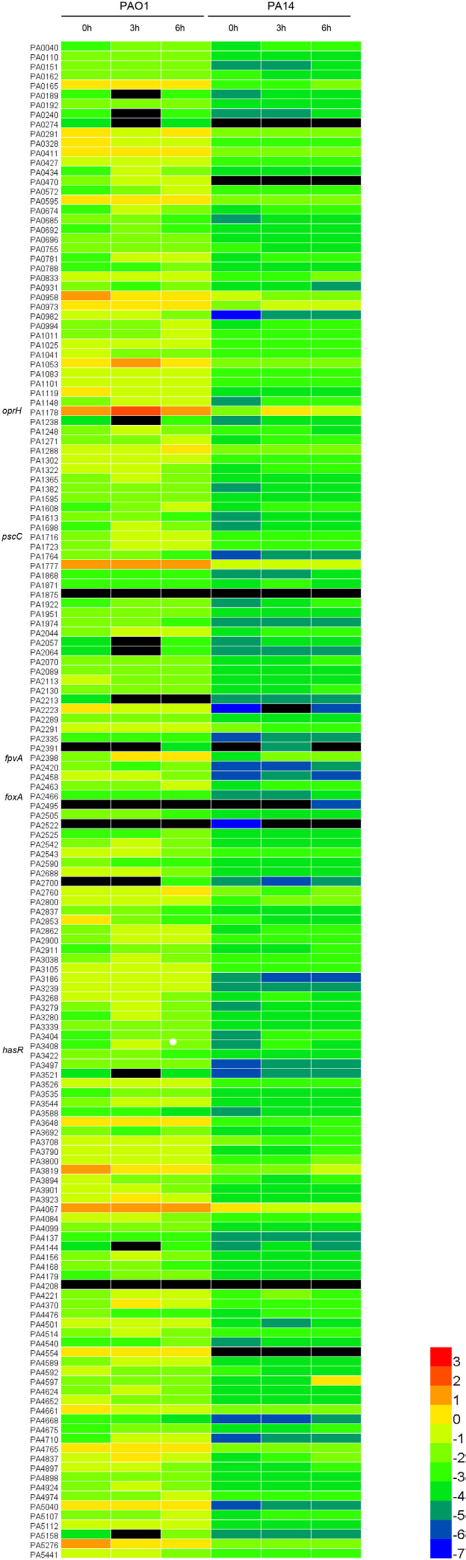
Expression levels of the outer membrane proteins relative to RpsL (ribosomal protein, serves as the internal control). The heatmap analysis was performed to illustrate the expression profile of the 158 outer membrane protein genes of PAO1 and PA14. The values are log base 10 of the relative expression levels. The black color represents genes undetected in quantitative real-time PCR.

To determine the expression levels of outer membrane proteins during infection, we designed quantitative real-time PCR primers for the 158 outer membrane protein genes in the *P. aeruginosa* PAO1 genome (Table S1 in Supplementary Material). Previously, Howell et al. (32) demonstrated that timely expression of T3SS genes at early stages of infection is essential for bacterial pathogenesis in the acute pneumonia model. And in this model, mice can die as early as 12 h postinfection. Therefore, we focused on early time points in our study. Each mouse was infected with 1 × 10^9^ CFU of PAO1 intranasally. 3 or 6 h postinfection, BALF was obtained from at least six mice and pooled together. Bacteria from BALF were isolated, followed by RNA isolation and quantitative real-time PCR with the 16S RNA protein gene *rpsL* as an internal control. In order to identify proteins with common expression patterns in different *P. aeruginosa* strain backgrounds, we performed the infection experiment with another widely used wild-type strain PA14 (5 × 10^8^ CFU per mouse), which belongs to a different serogroup and is more virulent than PAO1 (23). The relative expression level of each protein is shown in Figure 1 as a heatmap and also presented as percentage of the internal control in Table S2 in Supplementary Material.

**Figure 2:**
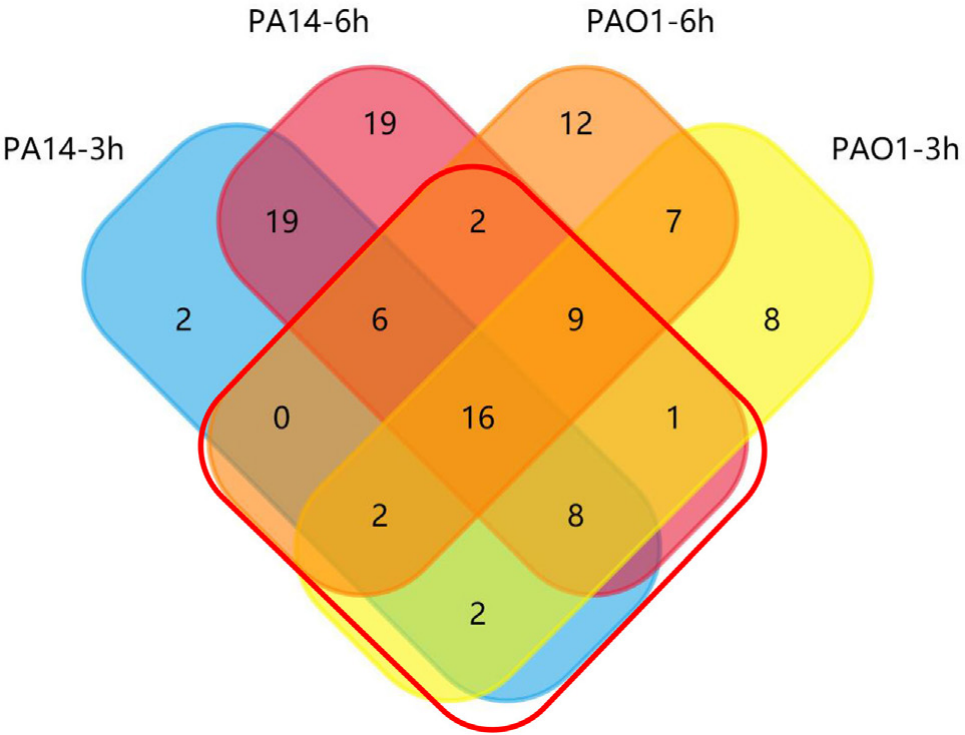
Venn diagram of upregulated outer membrane genes during lung infection. Distribution of induced genes (fold change ≥ 2.0 relative to *in vitro* grown bacteria) in PAO1 and PA14 at 3 and 6 h postinfection. Genes in the red frame were selected for further screening.

To identify genes highly expressed during infection (fold change ≥ 2.0), we performed pair-wise comparisons to determine the relative gene expression levels during infection. Four comparisons, PAO1 or PA14 grown *in vitro* versus the isogenic strain isolated from BALF 3 or 6 h postinfection, were each designated as PAO1 3 h, PAO1 6 h, PA14 3 h, and PA14 6 h, respectively. This led to the identification of 53, 54, 55, and 80 upregulated genes for the PAO1 3 h, PAO1 6 h, PA14 3 h, and PA14 6 h, respectively. To identify genes that were highly expressed commonly in both PAO1 and PA14 during infection, the data of the pair-wise comparisons were plotted using a four-way Venn diagram (Figure 2). Of all the genes tested, 46 were found highly expressed in both PAO1 and PA14 during infection, i.e., in all the four conditions (Table S2 in Supplementary Material, highlighted in yellow). Besides, 13 genes were upregulated more than 10-fold at least in one of the four conditions (Table S2 in Supplementary Material, highlighted in red). In combination, 59 genes were selected for further testing in samples from individual mice rather than pooled samples. To confirm the expression levels of the selected genes in individual mice, we performed the infection again and purified the bacterial RNA from BALF from each infected mouse. The expression levels of the 59 genes are listed in Table S3 in Supplementary Material. Of these, 34 genes consistently show high-level expression during infection (Table S4 in Supplementary Material).

After the three round of screening, we found that *oprH* is one of the most highly expressed genes at all the conditions (Table S4 in Supplementary Material). Previously, it was reported that *oprH* is highly induced when *P. aeruginosa* interacts with epithelia cells (33). Recent studies demonstrated that OprH is a binding target of mammalian surfactant protein A (34) and C3 complement (35), indicating accessibility by host factors. In addition, high levels of antibodies against OprH were identified in young children with cystic fibrosis (36), indicating its high immunogenicity. These results suggest that immunization with OprH might elicit opsonic antibodies. Therefore, based on its expression level, accessibility, and immunogenicity, we selected OprH as a vaccine candidate.

### Further Identification of Potential Vaccine Candidates by Studying the Roles of Highly Expressed Outer Membrane Proteins in *P. aeruginosa* Virulence

**Figure 3:**
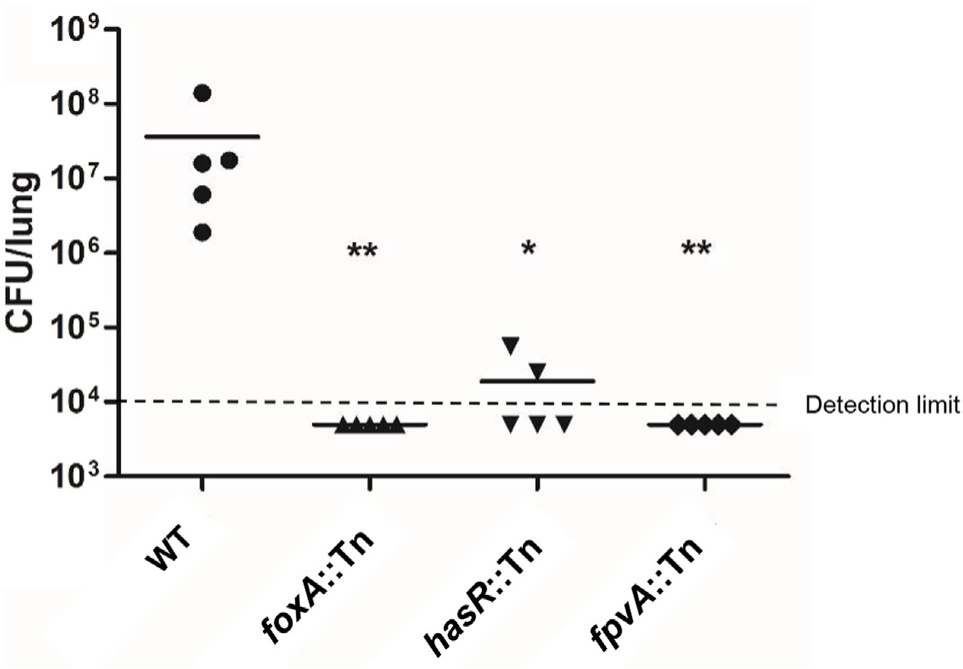
Bacterial colonization in the murine acute pneumonia model. Each mouse was infected by 2 × 10^7^ CFU of the indicated strain intranasally. 12 h after infection, mice were sacrificed and bacterial loads in the lungs were determined. Bacterial loads in mice infected with wild-type PA14 and the *oprH*:Tn, *foxA*:Tn, *hasR*:Tn, and *fpvA*:Tn. Bars represent medians, and error bars represent SEM. *p < 0.05; **p < 0.01 compared to wild-type PA14 by the Kruskal–Wallis with Dunn’s multiple comparison test. NS, not significant.

As antibodies that block the function of critical virulence factors might confer protection against bacterial pathogens (37), we evaluated the roles of the 34 highly expressed genes in bacterial virulence to identify more vaccine candidates. Transposon (Tn) mutants for each of the genes were picked from a nonredundant Tn insertion mutant library in the PA14 background (24). Among the 34 selected genes, three were associated with the T3SS, including *popN, pscC*, and *pscJ*. It has been well established that the T3SS plays an essential role in *P. aeruginosa* virulence in the acute pneumonia model (38–41). Thus, we utilized a *pscC*:Tn mutant as a control for attenuated virulence. Since OprF has been shown to contribute to bacterial virulence (42) and used in vaccine construction, we did not include it in our further testing. The *algE* mutant was not available in the library. In total, 30 Tn insertion mutants were tested for colonization in the murine acute pneumonia model. Consistent with previous studies (43), the *pscC*:Tn mutant was highly attenuated (Figure S1 in Supplementary Material). Mutation in the iron acquisition receptor genes (44), including *fpvA, foxA*, and *hasR*, significantly reduced bacterial loads (Figure 3). No drastic reductions in bacterial loads were observed in the other Tn insertion mutants (Figure S1 in Supplementary Material). Based on these observations, we suspected that antibodies against FpvA, FoxA, and HasR might block the functions of these proteins, thus reducing bacterial virulence. Overall, FpvA, FoxA, HasR, and OprH were chosen for vaccine construction.

### Protective Efficacy of the Vaccine Based on Iron Uptake Proteins

**Figure 4:**
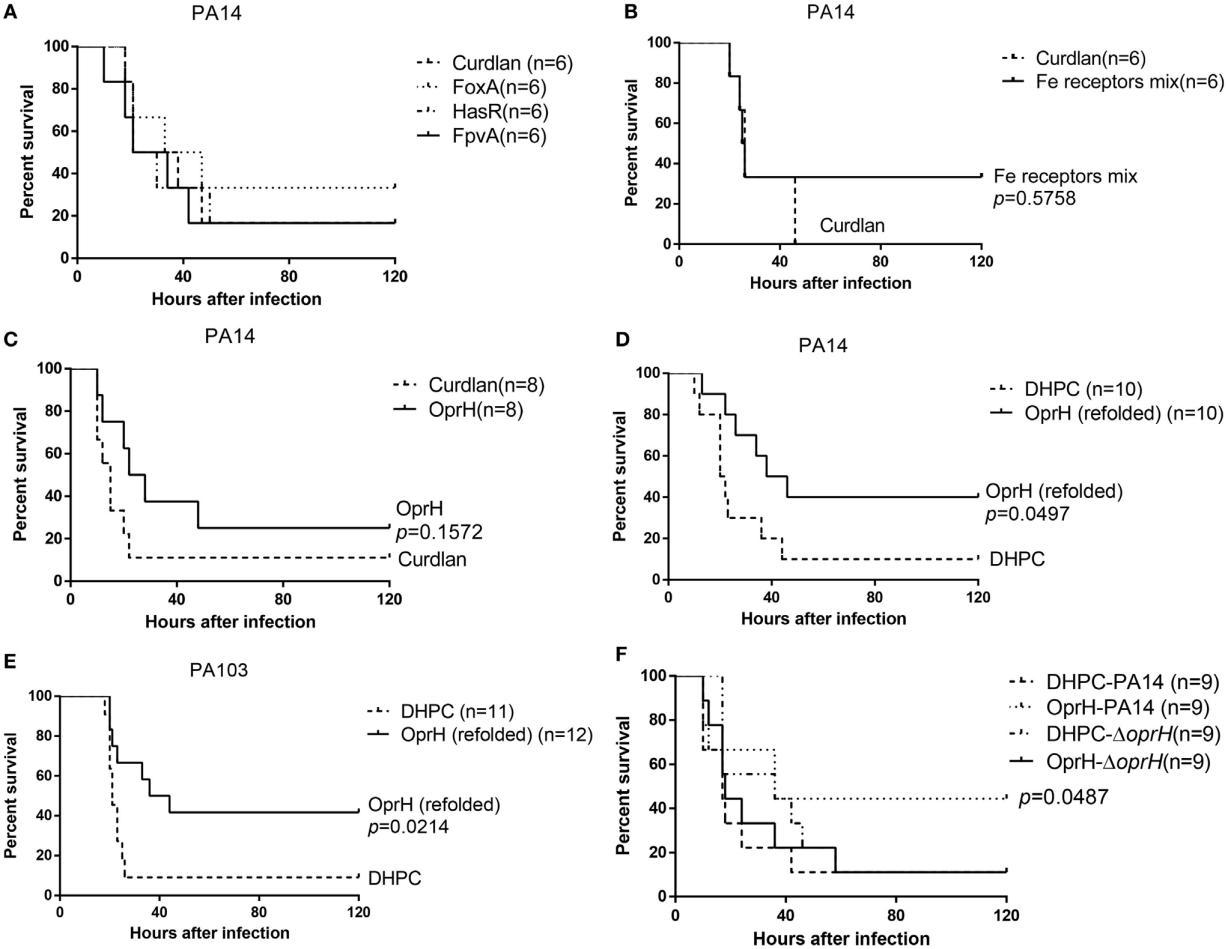
Survival rates of immunized mice. Mice were immunized with FpvA, HasR, and FoxA individually **(A)**, the mixture of purified FpvA, HasR, and FoxA **(B)**, or purified OprH **(C)**. Curdlan was used as the adjuvant. The immunized mice were challenged with wild-type PA14 (2 × 10^7^ CFU per mouse) intranasally. **(D)** Mice immunized with OprH refolded in DHPC with curdlan or curdlan alone were challenged with wild-type PA14 (2 × 10^7^ CFU per mouse) or PA103 (2 × 10^6^ CFU per mouse) **(E)** or the PA14 Δ*oprH* strain **(F)**. The mice were monitored for 5 days. *p* Values were calculated by log-rank test. The *p* value in **(F)** is the comparison between PA14 or the Δ*oprH* challenged OprH immunized mice.

Previously, we found that FpvA could elicite Th17 response in mice (31). To examine the protective efficacies of vaccines based on the iron acquisition receptor proteins, 6× His-tagged FpvA, HasR, or FoxA was individually overexpressed in *E. coli* and purified under denatured condition with Ni-NTA (Figure S2A in Supplementary Material). Each protein was further purified by molecular sieve. Each of the purified FpvA, HasR, and FoxA was mixed with curdlan (20 mg/mL), which has been shown to increase IgG titers (45, 46). The final concentrations of FpvA, HasR, and FoxA were 0.1, 0.1, and 0.25 mg/mL, respectively. Each mouse was immunized intranasally with 20 µL of the individual proteins. Compared with curdlan along, FpvA, HasR, and FoxA immunization did not show protective efficacy in the murine acute pneumonia model (Figure 4A).

We suspected that the individual protein might not elicit sufficient antibodies. Therefore, we constructed a trivalent vaccine by combining FpvA, HasR, and FoxA. Equal amount of His-FpvA, His-HasR, and His-FoxA were mixed together and dialyzed into PBS, resulting in a final concentration of 0.9 mg/mL. The proteins were mixed with same volume of curdlan. Each mouse was immunized intranasally with 20 µL of the mixture. Compared with curdlan along, the trivalent vaccine did not protect mice in the acute pneumonia model (Figure 4B).

### Protective Efficacy of OprH Vaccination

The His-OprH was purified under denatured condition with Ni-NTA (Figure S2B in Supplementary Material), followed by sequential dialysis in PBS with reducing concentrations of urea. Afterward, OprH at 1 mg/mL was mixed with equal volume of curdlan (20 mg/mL) and each mouse was immunized intranasally with 20 µL of the mixture, resulting in 10 µg of OprH per mouse. Compared with curdlan alone, the OprH vaccine did not show significantly protective efficacy in the acute pneumonia model (Figure 4C).

Previously, Edrington et al. (26) revealed the structure of OprH by refolding the purified protein in DHPC micelles. Refolding of OprH greatly increased its solubility. More importantly, outer membrane proteins with their natural conformation, as would be expected in DHPC micelles, may be more likely to elicit opsonic antibodies. Therefore, we utilized refolded His-OprH in DHPC micelles with curdlan in vaccination. As reported previously, the efficiency of OprH refolding could be monitored by SDS-PAGE (26). The apparent molecular mass of OprH on the SDS-PAGE gel would change when the proteins were transferred from an unfolded to a folded form. The refolded protein runs at 18 kDa but shifts to 21 kDa after boiling in SDS-PAGE loading buffer (Figure S3 in Supplementary Material). This reversible “heat modifiability” indicated that the OprH was refolded successfully.

The highest soluble concentration of the refolded OprH was 7 mg/mL. The protein suspension was mixed with equal volume of curdlan and 20 µL of the mixture was used to immunize each mouse, resulting in 70 µg refolded OprH per mouse. Vaccination with OprH resulted in 40% survival after lung challenge with PA14 (serogroup O19), whereas vaccination with DHPC resulted in 10% survival (Figure 4D). PA103 (serogroup O11) is a highly virulent clinical isolate. As shown in Figure 4E, mice immunized with OprH had more than 40% survival after challenge with PA103, compared with 10% survival of those immunized with DHPC alone. To confirm that the protection was due to OprH-specific antibodies, the immunized mice were challenged with an *oprH* deletion mutant of PA14. As shown in Figure 4F, immunization with the refolded OprH was unable to confer protection against the Δ*oprH* mutant.

### Subtype of Induced Immunoglobulin in Lungs of OprH Immunized Mice

To the subtype of immunoglobulin induced by the OprH immunization in the lungs, BALF was obtained from mice 3 weeks after the final immunization, followed by ELISAs. The plates were coated with purified His-OprH fusion protein. BALF from immunized mice was used as the primary antibody, and goat anti-mouse IgG antibody or goat anti-mouse IgA antibody was used as the secondary antibody (Figure S5 in Supplementary Material). OprH-specific IgG was detected in the BALFs of OprH immunized mice, whereas no IgA antibody was detected.

### Antigen-Specific Serum Antibodies From Iron Uptake Proteins and OprH Immunized Mice

**Figure 5:**
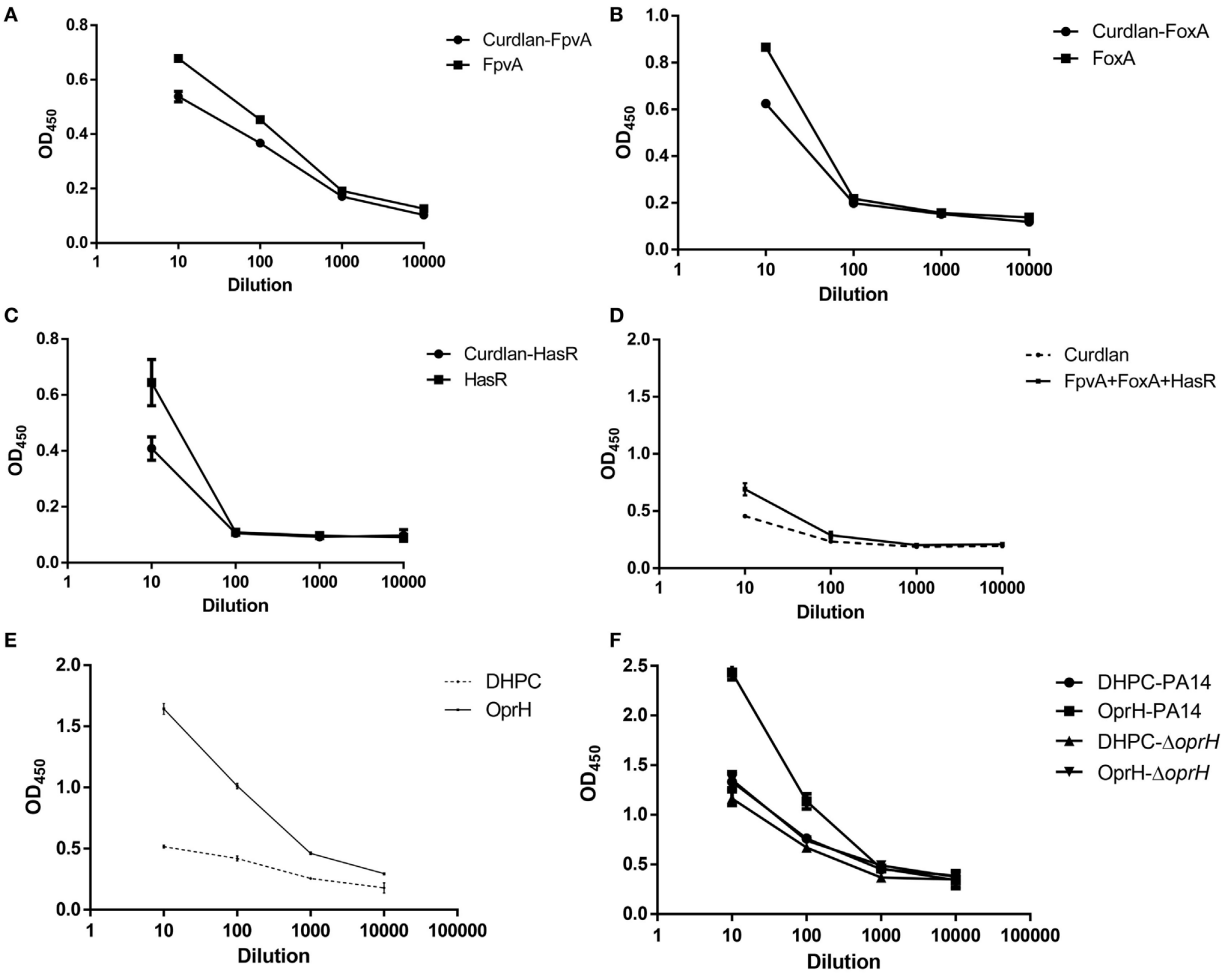
Enzyme linked immunosorbent assay (ELISA) of iron uptake proteins or OprH. **(A–D)** FpvA, FoxA, HasR, or mixture of iron uptake proteins were used to coat plates. The ELISAs were conducted using sera from mice immunized with each of the iron uptake proteins or the combination of these proteins with curdlan or curdlan alone. **(E)** Sera from mice immunized with the refolded OprH or DHPC alone with curdlan were tested for antigen-specific IgG levels by ELISA with plates coated with OprH. **(F)** ELISA of the whole bacterial cells. The cells of PA14 or the Δ*oprH* mutant were coated on an ELISA plate. Sera from mice immunized with refolded OprH or DHPC alone with curdlan were tested for the levels IgG that can bind to the cells by ELISA. Each point is the average of duplicates using pooled sera from five to seven mice, and error bars are SDs.

To test the humoral immune responses elicited by immunization with iron uptake proteins and OprH, sera from immunized mice were collected 3 weeks after the third immunization and the antigen-specific IgG titers were determined by ELISA. Plates were coated with the purified FpvA, FoxA, and HasR, the mixture of these three proteins or the OprH without refolding. Then sera from immunized mice were used as the primary antibody. Immunization with FpvA, FoxA, HasR, and the combination of the three proteins elicited minimal antigen-specific IgG (Figures 5A–D), whereas immunization with the refolded OprH elicited a high level of antigen-specific IgG (Figure 5E). ELISA of whole bacterial cells was also conducted to investigate if the elicited antibodies toward OprH can bind with the OprH on the bacterial surface. As shown in Figure 5F, the antibodies from OprH immunized mice bound with wild-type PA14, but not the Δ*oprH* mutant. These results indicate that immunization with the refolded OprH elicited antibodies that recognize the OprH exposed on the bacterial surface.

### Antibody-Mediated Phagocytosis of Bacteria by BMDM

**Figure 6:**
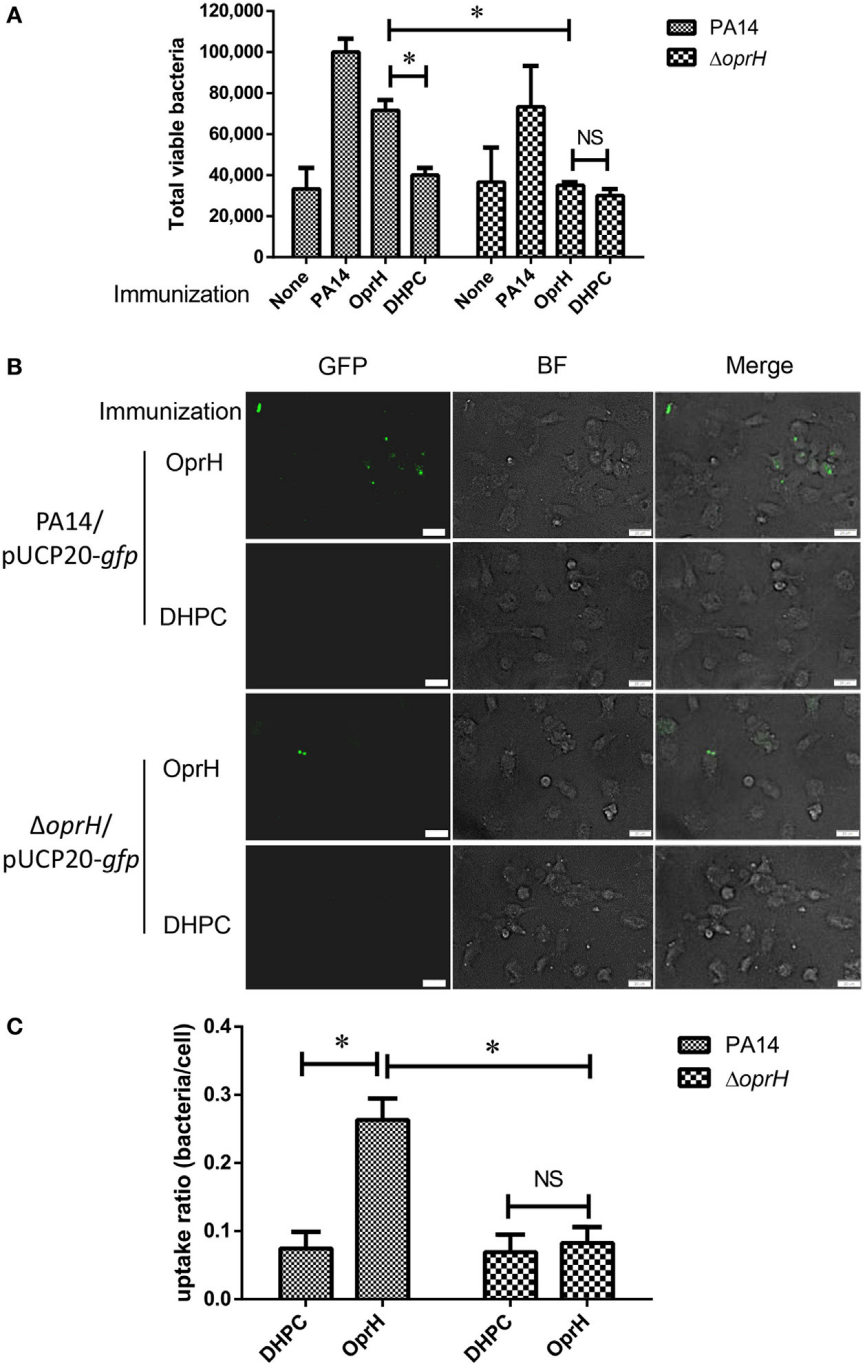
Opsonophagocytic uptake by BMDM in the presence of sera from immunized mice. **(A)** Opsonophagocytic uptake assays with wild-type PA14 and the Δ*oprH* mutant. Bacteria were premixed with sera from immunized mice and then incubated with BMDM. Values represent the mean total viable colonies captured by BMDM from three assay replicates for each sample. **(B)** Fluorescent bacteria (PA14/pUCP20-*gfp* and Δ*oprH*/pUCP20-*gfp*) were subjected to the opsonophagocytic uptake assay. The uptaken bacteria were observed by fluorescent microscope. **(C)** The uptake ratio of bacteria by BMDM cells was calculated by counting cells in at least three fields of each sample. Abbreviations: BF, bright field; BMDM, bone marrow-derived macrophage.

We next performed phagocytosis assay with BMDM in the presence of sera from mice immunized with refolded His-OprH, DHPC, uninfected mice, and those infected with PA14. The highest capture of PA14 cells was observed in the presence of sera from PA14 infected mice. The sera from His-OprH immunized mice enabled more uptake than those from DHPC-immunized mice (Figure 6A). The difference was abolished when a Δ*oprH* mutant was used in the assay, indicating a specificity of the antibodies from His-OprH immunized mice (Figure 6A).

To visualize the phagocytic uptake, BMDMs were incubated with PA14 and the Δ*oprH* mutant overexpressing GFP. The presence of sera from His-OprH-immunized mice induced more phagocytosis of PA14 than those from DHPC-immunized mice, whereas no difference was observed on the Δ*oprH* mutant (Figures 6B,C). However, the sera from mice immunized with the mixture of FpvA, FoxA, and HasR were unable to promote phagocytosis (Figure S4 in Supplementary Material). Overall, these results indicate that immunization with the refolded His-OprH induced phagocytic antibodies that specifically targeting bacterial surface OprH.

## Discussion

Lipopolysaccharide O antigen-based vaccines have been found to confer effective protection against *P. aeruginosa* in animal models (6), which might be due to the high abundance and accessibility by antibodies. However, the narrow protection spectrum (same LPS serogroup) impedes the application of this type of vaccine (47, 48). Compared to the highly variable O antigens, outer membrane proteins are relatively more conserved. Therefore, vaccines based on these proteins may induce antibodies against a broad range of *P. aeruginosa* serotypes.

Antibodies recognizing the surface protein or structures might promote opsonophagocytic killing by phagocytes, which plays an important role in the protection against bacterial infection (49). In addition, it has been demonstrated that antibodies against the T3SS needle protein PcrV were able to protect cells from T3SS-mediated cytotoxicity (37). And the F(ab′)2 fragment of the PcrV antibody was able to neutralized the T3SS and protect mice against *P. aeruginosa* infection in an Fc fragment-independent manner (50). These results demonstrate that an antibody targeting a virulence factor might confer protection by blocking the function of the virulence factor independent of the Fc-mediated phagocytosis.

Besides humoral immune responses, Th17 responses have been shown to play an important role in host defense against a variety of pathogens (51). Previously, we found that immunization with the T3SS component PopB conferred protection in mice in a Th17-dependent manner (31). However, the elicited antibodies could not mediate opsonophagocytic killing or block T3SS-mediated cytotoxicity. Ideally, a combination of Th17 responses and protective antibodies will likely confer the broadest and most potent protection.

In order to identify novel antigens to construct mono- or multi-valent vaccines, here we examined the expression profiles of all the *P. aeruginosa* outer membrane proteins during infection. We found that the expression of OprH was highly induced during infection (Table S4 in Supplementary Material). Initially, purified OprH was directly used in immunization. However, no significant protection was observed in the murine acute pneumonia model. We suspected that one of the major causes is that the membrane localized OprH without its natural conformation might not be able to elicit effective opsonic antibody. To regain the natural conformation of OprH on bacterial surface, we took advantage of the DHPC refolding method, which had been used to solve the structure of OprH (26). In addition, the refolding increased the concentration of soluble OprH, enabling higher amount of protein used in immunization. Antibodies elicited by the refolded OprH induced phagocytic uptake by BMDM (Figure 6), indicating recognition of the surface exposed domains of OprH, which was further confirmed by ELISA with whole bacterial cells (Figure 5F). In our study, intranasal immunization might elicit specific IgA, which might protect the mucosal surface. However, the IgA level was low in the OprH immunized mice (Figure S5 in Supplementary Material). Since the elicited IgG binds to the bacterial surface OprH and promote phagocytosis of the bacteria, the refolded OprH might be a potential vaccine candidate. Other adjuvants and immunization routes, such as aluminum adjuvant and subcutaneous injection might increase the antibody titer and protective efficacy. Further studies are needed to evaluate the effects. In addition, vaccination with multiple *P. aeruginosa* proteins has been demonstrated to confer protection in various mouse infection models (9, 31, 49, 52, 53), including OprF, OprI, flagellin, PcrV, PopB, etc. OprF, OprI, and flagellin elicit opsonic antibodies. And antibodies against PcrV protect cells from T3SS-mediated cytotoxicity. Therefore, fusion proteins of OprH with these above proteins might induce antibodies against multiple targets thus increase the protective efficacy. Since immunization with PopB elicits Th17 response, which has been shown to be important in the protection against *P. aeruginosa* (31), an OprH and PopB fusion protein might also increase the protective efficacy.

Previous studies and our results here demonstrated that FpvA was highly upregulated during infection (Table S4 in Supplementary Material) (54). FpvA is the receptor of pyoverdine, which is a major iron acquisition molecule secreted by *P. aeruginosa* under iron-limiting conditions (54). In addition, FpvA is involved in the regulation of multiple virulence factors, such as pyoverdine, exotoxin A, and PrpL endoprotease (55). Another two iron acquisition proteins HasR and FoxA are also required for the bacterial virulence [(56) and our study here]. Therefore, antibodies against FpvA, HasR, and FoxA might promote opsonic phagocytosis, block iron acquisition, and repress bacterial virulence, which makes them promising vaccine candidates. However, immunization with FpvA, HasR, and FoxA did not confer protection against lung infection. After immunization of mice, iron uptake proteins did not elicit protective antibodies (Figures 5A–D), which might be due to the poor immunogenicity of the denatured proteins or insufficient amount of antigens. Refolding these proteins in liposomes might elicit antibodies recognizing the exposed portion of them. Numerous detergents and lipids have been used in protein refolding, such as dodecylphoshocholine, 1-myristoyl-2-hydroxy-*sn*-glycero-3-phosphocholine, *N,N*-dimethyldodecylamine-*N*-oxide, DHPC, etc. Further studies are required to find out the optimum *in vitro* refolding conditions and biochemical analysis and NMR examinations are required to test whether the proteins are folded in the natural structure (26). Another option is to overexpress the proteins in attenuated *P. aeruginosa* or *Salmonella*.

Overall, we developed a strategy to identify potential vaccine candidates and demonstrated protection against *P. aeruginosa* lung infection by intranasal immunization with refolded OprH. Combination of OprH with other antigens might further increase the protective efficacy and thus warrants further study.

## Ethics Statement

All animal studies complied with National and Nankai University guidelines regarding the use of animals in research. All animal experiment protocols have been approved by the institutional animal care and use committee of the College of Life Sciences of Nankai University (permit number NK-04-2012).

## Author Contributions

Conceived and designed the experiments: WW, CL, SJ, and GP. Performed the experiments: CL, XP, BX, and FC. Analyzed the data: CL, WW, SJ, YJ, FB, ZC, and GP. Wrote the paper: CL, WW, and SJ.

## Conflict of Interest Statement

The authors declare that the research was conducted in the absence of any commercial or financial relationships that could be construed as a potential conflict of interest.

## Supplementary Material

Figure S1 Colonization of indicated strains in the murine acute pneumonia model. Mice were infected by the bacteria intranasally. 12 h postinfection, the bacterial loads in the lungs were determined by plating. Bars represent medians, and error bars represent SEM.

Figure S2 Coomassie brilliant blue staining after SDS-PAGE of purified proteins. **(A)** SDS-PAGE of the purified His-HasR, His-FoxA, and His-FpvA. **(B)** SDS-PAGE of the purified N-terminal 6× His-tagged OprH.

Figure S3 SDS-PAGE analysis of the refolded OprH with or without boiling in the SDS-PAGE loading buffer.

Figure S4 Opsonophagocytic uptake assays with wild-type PA14. Bacteria were premixed with sera from mice immunized with the mixture of the iron acquisition proteins and then incubated with bone marrow-derived macrophage (BMDM). Values represent the mean total viable colonies captured by BMDM from three replicates for each sample.

Figure S5 Enzyme linked immunosorbent assay (ELISA) of OprH to determine the subtype of immunoglobulin induced by OprH in lungs of immunized mice. Three weeks after the last immunization, bronchoalveolar lavage fluids (BALFs) were collected from the immunized mice and the antibodies against OprH were examined by ELISA. Each point is the average of duplicates using pooled BALFs from five mice, and error bars are SDs.

Figure S6 Binding of C3 to OprH in the presence of antibodies against OprH. **(A)** The purified OprH was run on SDS-PAGE gel and transferred to a PVDF membrane. The membrane was incubated with heat-inactivated sera (1:2,000) from mice immunized by DHPC or OprH for 1 h at room temperature. After washing with PBST for four times, the membrane was incubated with human serum (1:500 dilution) for 30 min. Then the membrane was incubated with a rabbit anti-C3 antibody. **(B)** Western blotting was conducted with the same amount of OprH samples with an anti-His antibody. After exposure, the average intensity of each band was calculated by ImageJ. The value of the sample incubated with human sera was divided by the value of that probed with the anti-His antibody **(C)**.

Table S1 RT-PCR primers.

Table S2 Relative expression levels of outer membrane proteins.

Table S3 Relative gene expression levels in individual mouse.

Table S4 Expression levels of selected genes relative to *rpsL* (internal control).

## References

[1] Thirumala R Ramaswamy M Chawla S. Diagnosis and management of infectious complications in critically ill patients with cancer. Crit Care Clin (2010) 26(1):59–91. 10.1016/j.ccc.2009.09.007 19944276

[2] Talwalkar JS Murray TS. The approach to *Pseudomonas aeruginosa* in cystic fibrosis. Clin Chest Med (2016) 37(1):69–81. 10.1016/j.ccm.2015.10.004 26857769

[3] Brooun A Liu S Lewis K. A dose-response study of antibiotic resistance in *Pseudomonas aeruginosa* biofilms. Antimicrob Agents Chemother (2000) 44(3):640–6. 10.1128/AAC.44.3.640-646.2000 10681331 PMC89739

[4] Lister PD Wolter DJ Hanson ND. Antibacterial-resistant *Pseudomonas aeruginosa*: clinical impact and complex regulation of chromosomally encoded resistance mechanisms. Clin Microbiol Rev (2009) 22(4):582–610. 10.1128/CMR.00040-09 19822890 PMC2772362

[5] Priebe GP Goldberg JB. Vaccines for *Pseudomonas aeruginosa*: a long and winding road. Expert Rev Vaccines (2014) 13(4):507–19. 10.1586/14760584.2014.890053 24575895 PMC4521563

[6] Pier GB. Promises and pitfalls of *Pseudomonas aeruginosa* lipopolysaccharide as a vaccine antigen. Carbohydr Res (2003) 338(23):2549–56. 10.1016/S0008-6215(03)00312-4 14670716

[7] Sawa T Ito E Nguyen VH Haight M. Anti-PcrV antibody strategies against virulent *Pseudomonas aeruginosa*. Hum Vaccin Immunother (2014) 10(10):2843–52. 10.4161/21645515.2014.971641 25483637 PMC5443083

[8] Krause A Whu WZ Qiu J Wafadari D Hackett NR Sharma A RGD capsid modification enhances mucosal protective immunity of a non-human primate adenovirus vector expressing *Pseudomonas aeruginosa* OprF. Clin Exp Immunol (2013) 173(2):230–41. 10.1111/cei.12101 23607394 PMC3722923

[9] Campodonico VL Llosa NJ Grout M Doring G Maira-Litran T Pier GB. Evaluation of flagella and flagellin of *Pseudomonas aeruginosa* as vaccines. Infect Immun (2010) 78(2):746–55. 10.1128/IAI.00806-09 19995892 PMC2812208

[10] Banadkoki AZ Keshavarzmehr M Afshar Z Aleyasin N Fatemi MJ Behrouz B Protective effect of pilin protein with alum+naloxone adjuvant against acute pulmonary *Pseudomonas aeruginosa* infection. Biologicals (2016) 44(5):367–73. 10.1016/j.biologicals.2016.06.009 27427517

[11] Haghbin M Armstrong D Murphy ML Controlled prospective trial of *Pseudomonas aeruginosa* vaccine in children with acute leukemia. Cancer (1973) 32(4):761–6. 10.1002/1097-0142(197310)32:4<761::AID-CNCR2820320405>3.0.CO;2-H 4201654

[12] Pennington JE Reynolds HY Wood RE Robinson RA Levine AS. Use of a *Pseudomonas aeruginosa* vaccine in patients with acute leukemia and cystic fibrosis. Am J Med (1975) 58(5):629–36. 10.1016/0002-9343(75)90498-2 805535

[13] Weimer ET Ervin SE Wozniak DJ Mizel SB. Immunization of young African green monkeys with OprF epitope 8-OprI-type A- and B-flagellin fusion proteins promotes the production of protective antibodies against nonmucoid *Pseudomonas aeruginosa*. Vaccine (2009) 27(48):6762–9. 10.1016/j.vaccine.2009.08.080 19744586

[14] Hassan R El-Naggar W Abd El-Aziz AM Shaaban M Kenawy HI Ali YM Immunization with outer membrane proteins (OprF and OprI) and flagellin B protects mice from pulmonary infection with mucoid and nonmucoid *Pseudomonas aeruginosa*. J Microbiol Immunol Infect (2018) 51(3):312–20. 10.1016/j.jmii.2016.08.014 28291719

[15] Rello J Krenn CG Locker G Pilger E Madl C Balica L A randomized placebo-controlled phase II study of a *Pseudomonas* vaccine in ventilated ICU patients. Crit Care (2017) 21(1):22. 10.1186/s13054-017-1601-9 28159015 PMC5291979

[16] DiGiandomenico A Keller AE Gao C Rainey GJ Warrener P Camara MM A multifunctional bispecific antibody protects against *Pseudomonas aeruginosa*. Sci Transl Med (2014) 6(262):262ra155. 10.1126/scitranslmed.3009655 25391481

[17] Baumann U Gocke K Gewecke B Freihorst J von Specht BU. Assessment of pulmonary antibodies with induced sputum and bronchoalveolar lavage induced by nasal vaccination against *Pseudomonas aeruginosa*: a clinical phase I/II study. Respir Res (2007) 8:57. 10.1186/1465-9921-8-57 17683588 PMC1973076

[18] Doring G Meisner C Stern M Flagella Vaccine Trial Study Group. A double-blind randomized placebo-controlled phase III study of a *Pseudomonas aeruginosa* flagella vaccine in cystic fibrosis patients. Proc Natl Acad Sci U S A (2007) 104(26):11020–5. 10.1073/pnas.0702403104 17585011 PMC1904125

[19] Francois B Luyt CE Dugard A Wolff M Diehl JL Jaber S Safety and pharmacokinetics of an anti-PcrV PEGylated monoclonal antibody fragment in mechanically ventilated patients colonized with *Pseudomonas aeruginosa*: a randomized, double-blind, placebo-controlled trial. Crit Care Med (2012) 40(8):2320–6. 10.1097/CCM.0b013e31825334f6 22622405

[20] Brunham RC Plummer FA Stephens RS Bacterial antigenic variation, host immune response, and pathogen-host coevolution. Infect Immun (1993) 61(6):2273–6. 8500868 10.1128/iai.61.6.2273-2276.1993 PMC280844

[21] Finlay BB Falkow S. Common themes in microbial pathogenicity revisited. Microbiol Mol Biol Rev (1997) 61(2):136–69. 9184008 10.1128/mmbr.61.2.136-169.1997 PMC232605

[22] Montor WR Huang J Hu Y Hainsworth E Lynch S Kronish JW Genome-wide study of *Pseudomonas aeruginosa* outer membrane protein immunogenicity using self-assembling protein microarrays. Infect Immun (2009) 77(11):4877–86. 10.1128/IAI.00698-09 19737893 PMC2772540

[23] Priebe GP Walsh RL Cederroth TA Kamei A Coutinho-Sledge YS Goldberg JB IL-17 is a critical component of vaccine-induced protection against lung infection by lipopolysaccharide-heterologous strains of *Pseudomonas aeruginosa*. J Immunol (2008) 181(7):4965–75. 10.4049/jimmunol.181.7.4965 18802100 PMC2597098

[24] Liberati NT Urbach JM Miyata S Lee DG Drenkard E Wu G An ordered, nonredundant library of *Pseudomonas aeruginosa* strain PA14 transposon insertion mutants. Proc Natl Acad Sci U S A (2006) 103(8):2833–8. 10.1073/pnas.0511100103 16477005 PMC1413827

[25] Clifford RJ Milillo M Prestwood J Quintero R Zurawski DV Kwak YI Detection of bacterial 16S rRNA and identification of four clinically important bacteria by real-time PCR. PLoS One (2012) 7(11):e48558. 10.1371/journal.pone.0048558 23139793 PMC3490953

[26] Edrington TC Kintz E Goldberg JB Tamm LK. Structural basis for the interaction of lipopolysaccharide with outer membrane protein H (OprH) from *Pseudomonas aeruginosa*. J Biol Chem (2011) 286(45):39211–23. 10.1074/jbc.M111.280933 21865172 PMC3234746

[27] Pier GB. Safety and immunogenicity of high molecular weight polysaccharide vaccine from immunotype 1 *Pseudomonas aeruginosa*. J Clin Invest (1982) 69(2):303–8. 10.1172/JCI110453 6799548 PMC370979

[28] Tennant SM Wang JY Galen JE Simon R Pasetti MF Gat O Engineering and preclinical evaluation of attenuated nontyphoidal *Salmonella* strains serving as live oral vaccines and as reagent strains. Infect Immun (2011) 79(10):4175–85. 10.1128/IAI.05278-11 21807911 PMC3187273

[29] Bridge DR Whitmire JM Gilbreath JJ Metcalf ES Merrell DS. An enterobacterial common antigen mutant of *Salmonella enterica* serovar Typhimurium as a vaccine candidate. Int J Med Microbiol (2015) 305(6):511–22. 10.1016/j.ijmm.2015.05.004 26070977

[30] Bridge DR Whitmire JM Makobongo MO Merrell DS. Heterologous *Pseudomonas aeruginosa* O-antigen delivery using a *Salmonella enterica* serovar Typhimurium wecA mutant strain. Int J Med Microbiol (2016) 306(7):529–40. 10.1016/j.ijmm.2016.06.005 27476047

[31] Wu W Huang J Duan B Traficante DC Hong H Risech M Th17-stimulating protein vaccines confer protection against *Pseudomonas aeruginosa* pneumonia. Am J Respir Crit Care Med (2012) 186(5):420–7. 10.1164/rccm.201202-0182OC 22723292 PMC3443805

[32] Howell HA Logan LK Hauser AR. Type III secretion of ExoU is critical during early *Pseudomonas aeruginosa* pneumonia. MBio (2013) 4(2):e00032–13. 10.1128/mBio.00032-13 23481600 PMC3604777

[33] Gellatly SL Needham B Madera L Trent MS Hancock RE. The *Pseudomonas aeruginosa* PhoP-PhoQ two-component regulatory system is induced upon interaction with epithelial cells and controls cytotoxicity and inflammation. Infect Immun (2012) 80(9):3122–31. 10.1128/IAI.00382-12 22710876 PMC3418734

[34] Qadi M Lopez-Causape C Izquierdo-Rabassa S Mateu Borras M Goldberg JB Oliver A Surfactant protein A recognizes outer membrane protein OprH on *Pseudomonas aeruginosa* isolates from individuals with chronic infection. J Infect Dis (2016) 214(9):1449–55. 10.1093/infdis/jiw387 27543671

[35] Qadi M Izquierdo-Rabassa S Mateu Borras M Domenech-Sanchez A Juan C Goldberg JB Sensing Mg2+ contributes to the resistance of *Pseudomonas aeruginosa* to complement-mediated opsonophagocytosis. Environ Microbiol (2017) 19(10):4278–86. 10.1111/1462-2920.13889 28805355

[36] Moore R Kyd JM Carzino R Armstrong D Grimwood K Otczyk DC Mucosal and systemic antibody responses to potential *Pseudomonas aeruginosa* vaccine protein antigens in young children with cystic fibrosis following colonization and infection. Hum Vaccin Immunother (2013) 9(3):506–14. 10.4161/hv.23226 23249482 PMC3891706

[37] Sawa T Yahr TL Ohara M Kurahashi K Gropper MA Wiener-Kronish JP Active and passive immunization with the *Pseudomonas* V antigen protects against type III intoxication and lung injury. Nat Med (1999) 5(4):392–8. 10.1038/7391 10202927

[38] Finck-Barbancon V Goranson J Zhu L Sawa T Wiener-Kronish JP Fleiszig SM ExoU expression by *Pseudomonas aeruginosa* correlates with acute cytotoxicity and epithelial injury. Mol Microbiol (1997) 25(3):547–57. 10.1046/j.1365-2958.1997.4891851.x 9302017

[39] Hauser AR Kang PJ Engel JN. PepA, a secreted protein of *Pseudomonas aeruginosa*, is necessary for cytotoxicity and virulence. Mol Microbiol (1998) 27(4):807–18. 10.1046/j.1365-2958.1998.00727.x 9515706

[40] Sawa T Ohara M Kurahashi K Twining SS Frank DW Doroques DB In vitro cellular toxicity predicts *Pseudomonas aeruginosa* virulence in lung infections. Infect Immun (1998) 66(7):3242–9. 9632591 10.1128/iai.66.7.3242-3249.1998 PMC108338

[41] Lee VT Smith RS Tummler B Lory S. Activities of *Pseudomonas aeruginosa* effectors secreted by the type III secretion system in vitro and during infection. Infect Immun (2005) 73(3):1695–705. 10.1128/IAI.73.3.1695-1705.2005 15731070 PMC1064929

[42] Fito-Boncompte L Chapalain A Bouffartigues E Chaker H Lesouhaitier O Gicquel G Full virulence of *Pseudomonas aeruginosa* requires OprF. Infect Immun (2011) 79(3):1176–86. 10.1128/IAI.00850-10 21189321 PMC3067511

[43] Smith RS Wolfgang MC Lory S. An adenylate cyclase-controlled signaling network regulates *Pseudomonas aeruginosa* virulence in a mouse model of acute pneumonia. Infect Immun (2004) 72(3):1677–84. 10.1128/IAI.72.3.1677-1684.2004 14977975 PMC356001

[44] Cornelis P. Iron uptake and metabolism in pseudomonads. Appl Microbiol Biotechnol (2010) 86(6):1637–45. 10.1007/s00253-010-2550-2 20352420

[45] LeibundGut-Landmann S Gross O Robinson MJ Osorio F Slack EC Tsoni SV Syk- and CARD9-dependent coupling of innate immunity to the induction of T helper cells that produce interleukin 17. Nat Immunol (2007) 8(6):630–8. 10.1038/ni1460 17450144

[46] Zygmunt BM Rharbaoui F Groebe L Guzman CA. Intranasal immunization promotes Th17 immune responses. J Immunol (2009) 183(11):6933–8. 10.4049/jimmunol.0901144 19890060

[47] Cryz SJ Jr Furer E Germanier R. Protection against fatal *Pseudomonas aeruginosa* burn wound sepsis by immunization with lipopolysaccharide and high-molecular-weight polysaccharide. Infect Immun (1984) 43(3):795–9. 6698608 10.1128/iai.43.3.795-799.1984 PMC264250

[48] Pier GB Thomas D Small G Siadak A Zweerink H. In vitro and in vivo activity of polyclonal and monoclonal human immunoglobulins G, M, and A against *Pseudomonas aeruginosa* lipopolysaccharide. Infect Immun (1989) 57(1):174–9. 2491835 10.1128/iai.57.1.174-179.1989 PMC313063

[49] Thanabalasuriar A Surewaard BG Willson ME Neupane AS Stover CK Warrener P Bispecific antibody targets multiple *Pseudomonas aeruginosa* evasion mechanisms in the lung vasculature. J Clin Invest (2017) 127(6):2249–61. 10.1172/JCI89652 28463232 PMC5451222

[50] Shime N Sawa T Fujimoto J Faure K Allmond LR Karaca T Therapeutic administration of anti-PcrV F(ab’)(2) in sepsis associated with *Pseudomonas aeruginosa*. J Immunol (2001) 167(10):5880–6. 10.4049/jimmunol.167.10.5880 11698464

[51] Rathore JS Wang Y. Protective role of Th17 cells in pulmonary infection. Vaccine (2016) 34(13):1504–14. 10.1016/j.vaccine.2016.02.021 26878294

[52] Chen TY Shang HF Chen TL Lin CP Hui CF Hwang J. Recombinant protein composed of *Pseudomonas* exotoxin A, outer membrane proteins I and F as vaccine against *P. aeruginosa* infection. Appl Microbiol Biotechnol (1999) 52(4):524–33. 10.1007/s002530051555 10570800

[53] Yang F Gu J Yang L Gao C Jing H Wang Y Protective efficacy of the trivalent *Pseudomonas aeruginosa* vaccine candidate PcrV-OprI-Hcp1 in murine pneumonia and burn models. Sci Rep (2017) 7(1):3957. 10.1038/s41598-017-04029-5 28638106 PMC5479855

[54] Folschweiller N Schalk IJ Celia H Kieffer B Abdallah MA Pattus F. The pyoverdin receptor FpvA, a TonB-dependent receptor involved in iron uptake by *Pseudomonas aeruginosa* (review). Mol Membr Biol (2000) 17(3):123–33. 10.1080/09687680050197356 11128971

[55] Beare PA For RJ Martin LW Lamont IL. Siderophore-mediated cell signalling in *Pseudomonas aeruginosa*: divergent pathways regulate virulence factor production and siderophore receptor synthesis. Mol Microbiol (2003) 47(1):195–207. 10.1046/j.1365-2958.2003.03288.x 12492864

[56] Minandri F Imperi F Frangipani E Bonchi C Visaggio D Facchini M Role of iron uptake systems in *Pseudomonas aeruginosa* virulence and airway infection. Infect Immun (2016) 84(8):2324–35. 10.1128/IAI.00098-16 27271740 PMC4962624

