# Supplementary figures and images for: Construction of a Protective Vaccine Against Lipopolysaccharide-Heterologous Pseudomonas aeruginosa Strains Based on Expression Profiling of Outer Membrane Proteins During Infection
Source: Front Immunol. 2018 Jul 26;9:1737. doi: 10.3389/fimmu.2018.01737 (PMC6070602; doi:10.3389/fimmu.2018.01737)

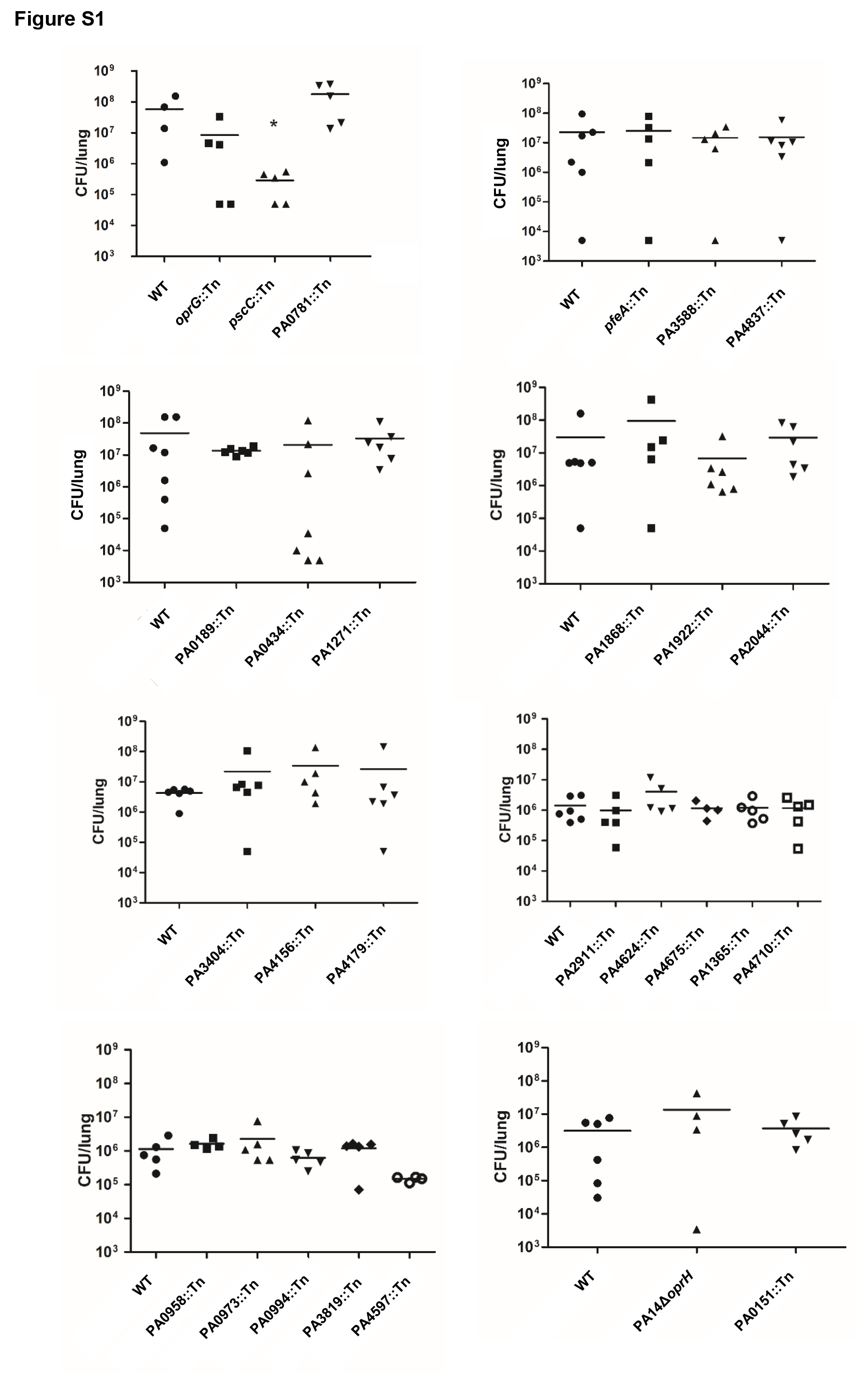

Supplement: Figure S1 — Colonization of indicated strains in the murine acute pneumonia model. Mice were infected by the bacteria intranasally. 12 h postinfection, the bacterial loads in the lungs were determined by plating. Bars represent medians, and error bars represent SEM. [file Image_1.tif]

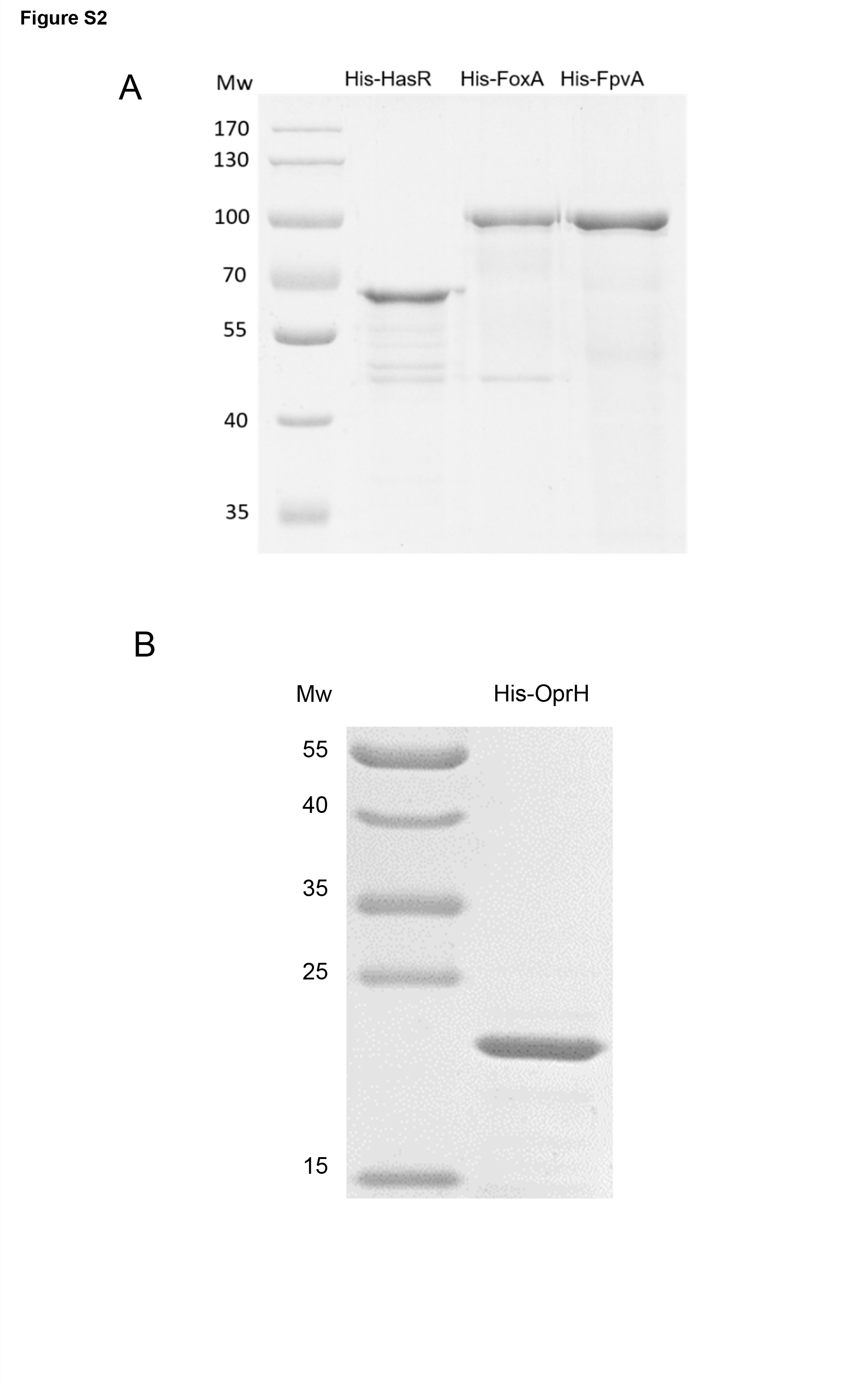

Supplement: Figure S2 — Coomassie brilliant blue staining after SDS-PAGE of purified proteins. (A) SDS-PAGE of the purified His-HasR, His-FoxA, and His-FpvA. (B) SDS-PAGE of the purified N-terminal 6× His-tagged OprH. [file Image_2.tif]
